# Testing the proficiency to distinguish locations with elevated plantar pressure within and between professional groups of foot therapists

**DOI:** 10.1186/1471-2474-7-93

**Published:** 2006-12-01

**Authors:** Nick A Guldemond, Pieter Leffers, Fred HM Nieman, Antal P Sanders, Nicolaas C Schaper, Geert HIM Walenkamp

**Affiliations:** 1 Orthopaedic Surgery, University Hospital Maastricht, P.O. Box 5800, 6202 AZ, Maastricht, The Netherlands; 2 Epidemiology, Faculty of Medicine, Maastricht University, P.O. Box 616, 6200 MD, Maastricht, The Netherlands; 3 Clinical Epidemiology and Medical Technology Assessment, University Hospital Maastricht, P.O. Box 5800, 6202 AZ, Maastricht, The Netherlands; 4 Rehabilitation Medicine, University Hospital Maastricht, P.O. Box 5800, 6202 AZ, Maastricht, The Netherlands; 5 Internal Medicine, University Hospital Maastricht, P.O. Box 5800, 6202 AZ, Maastricht, The Netherlands

## Abstract

**Background:**

Identification of locations with elevated plantar pressures is important in daily foot care for patients with rheumatoid arthritis, metatarsalgia and diabetes. The purpose of the present study was to evaluate the proficiency of podiatrists, pedorthists and orthotists, to distinguish locations with elevated plantar pressure in patients with metatarsalgia.

**Methods:**

Ten podiatrists, ten pedorthists and ten orthotists working in The Netherlands were asked to identify locations with excessively high plantar pressure in three patients with forefoot complaints. Therapists were instructed to examine the patients according to the methods used in their everyday clinical practice. Regions could be marked through hatching an illustration of a plantar aspect. A pressure sensitive platform was used to quantify the dynamic bare foot plantar pressures and was considered as 'Gold Standard' (GS). A pressure higher than 700 kPa was used as cut-off criterion for categorizing peak pressure into elevated or non-elevated pressure. This was done for both patient's feet and six separate forefoot regions: big toe and metatarsal one to five. Data were analysed by a mixed-model ANOVA and Generalizability Theory.

**Results:**

The proportions elevated/non-elevated pressure regions, based on clinical ratings of the therapists, show important discrepancies with the criterion values obtained through quantitative plantar pressure measurement. In general, plantar pressures in the big toe region were underrated and those in the metatarsal regions were overrated. The estimated method agreement on clinical judgement of plantar pressures with the GS was below an acceptable level: i.e. all intraclass correlation coefficient's equal or smaller than .60. The inter-observer agreement for each discipline demonstrated worrisome results: all below .18. The estimated mutual agreements showed that there was virtually no mutual agreement between the professional groups studied.

**Conclusion:**

Identification of elevated plantar pressure through clinical evaluation is difficult, insufficient and may be potentially harmful. The process of clinical plantar pressure screening has to be re-evaluated. The results of this study point towards the merit of quantitative plantar pressure measurement for clinical practice.

## Background

Elevated plantar peak pressures are associated with a wide variety of foot impairments like rheumatoid arthritis, metatarsalgia and diabetes. Reduction or off-loading of plantar tissue stress is a commonly therapeutical concept for these conditions. Off-loading strategies could include padding and strapping therapy, foot orthoses, therapeutic footwear or total contact casts [1,2]. Identification of locations with elevated plantar pressures seems to be logical requisite for effective off-loading [1-7]. Information about locations with elevated plantar pressure can be obtained through physical examination and is based on e.g. callus formation, blisters, soft tissue quality, painful regions and deformities. Various techniques such as footprints and podoscopes are used to measure static and dynamic plantar pressure patterns [8-12]. Advanced pressure sensitive devices i.e. platforms and in-shoe devises, provide information about the location as well as the magnitude of plantar peak pressure [1,5]. In The Netherlands this electronic equipment is sparsely used in clinical settings and the aforementioned traditional techniques are yet essential tools for daily practice.

In The Netherlands, allied foot care is provided by podiatrists, pedorthists (orthopaedic shoe technicians) and orthotists (orthopaedic technicians) [13-15]. Pedorthists are specialized in foot orthosis therapy for orthopaedic shoe wear, whereas podiatrists and orthotists mainly provide foot orthoses for non-orthopaedic shoe wear. In general, orthotists take care of more severe disorders than podiatrists. Although each discipline has a specific focus on particular foot problems, all three disciplines use physical examination and footprints for identification of locations with elevated plantar pressure. In literature, peak pressure values similar or higher than 700 kPa are frequently considered as abnormal or elevated [16-23]. On the contrary, studies in healthy subjects show that peak pressures were significantly lower than 700 kPa [24-29].

The purpose of the present study was to evaluate the proficiency of podiatrists, pedorthists and orthotists, to distinguish locations with relatively elevated plantar pressures in patients with metatarsalgia.

## Methods

### Therapists

As representatives of their professional groups, ten podiatrists, ten pedorthists and ten orthotists from the southern part of The Netherlands were asked to construct foot orthoses for three patients with metatarsalgia[15]. Podiatrists were approached through telephone directories. Companies of pedorthists and orthotic workshops were approached through member lists of the professional associations. Each delegated between one and three therapists for the study. The median professional experience in years (range) was 7.5 (14), 16.5 (34) and 20 (30) respectively for podiatrists, pedorthists and orthotists.

### Patients

Three patients with metatarsalgia: 2 females of 60 and 61 years old and a 37 years old male, with fore foot complaints and elevated plantar peak pressures were selected from an orthopaedic outpatient clinic. The male patient had foot problems related to psoriatic arthritis. Additional details about the patients are given in table 1. Before the start of the study, patients were informed about all study procedures and their possible risks. The Research Ethical Committee of the University Hospital Maastricht approved the study.

### Procedure

**Figure 1:**
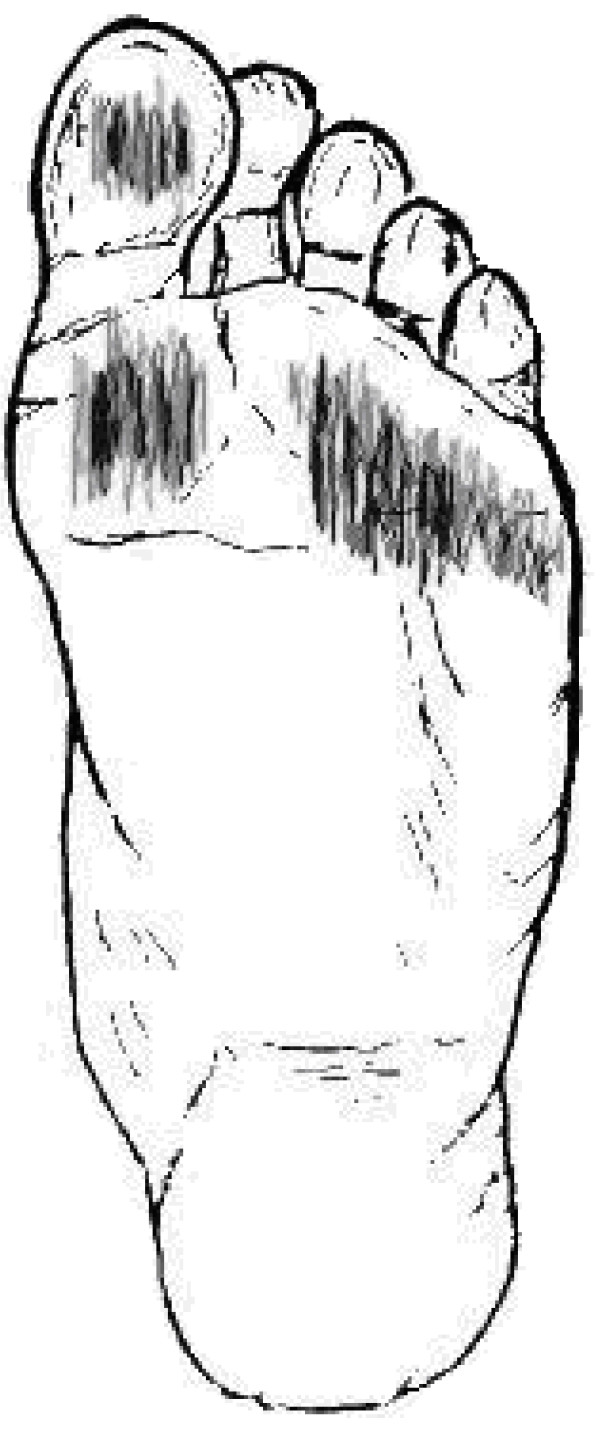
**Marking of regions with elevated plantar pressure**. Presumed regions with elevated plantar pressure are marked through hatching an illustration of a plantar aspect.

A central session was organised at the University Hospital Maastricht where the therapists could perform their physical examination for each patient. Therapists were asked to identify locations with elevated plantar pressure, which could be marked through hatching an illustration of a plantar aspect (figure 1). Therapists were instructed to examine the patients according to the methods used in their everyday clinical practice. An EMED SF-4^® ^pressure platform (Novel, Munich) was used to quantify the bare foot plantar pressures of the patient's feet and was performed according to a two-step protocol [30-34]. This measurement was considered as 'Gold Standard' (GS). Bare foot peak pressures were estimated per foot, by calculating the mean over the readings of 5 consecutive measurements. This was done for six discrete regions: big toe (BT) and metatarsal one (mt-1) to five (mt-5), demarcated by Novel 'create any mask^®^' software and verified through anterior-posterior radiographs[35].

### Statistical analysis

Data were analysed by a mixed-model ANOVA and Generalizability Theory[36]. The GS plantar peak pressure was categorized into two classes: 'non-elevated' i.e. less or equal to 700 kPa and 'elevated' i.e. more than 700 kPa. The six-factor ANOVA design was defined as '*Region *by *Side *within *Patient*' by '*Therapist *within *Group*' by '*Method*'. *Region *is a fixed factor with six levels: big toe and mt-1 to mt-5. *Side *is fixed with two levels: left or right foot. Both factors are nested within *Patient*, which can be seen as a random factor with three levels: subject A, B and C. *Therapist *is a random factor with ten levels, nested within *Group*, a fixed factor with the three levels of discipline: podiatrists, pedorthists and orthotists. Finally, *Method *is a fixed factor with two levels: proportions elevated/non-elevated pressure as rated by the therapists and proportions according to the GS. Proportions of relatively elevated plantar pressure, F-ratios with degrees of freedom and p-values belonging to the fixed part of the model, are computed with the computer program GENOVA[37].

Variance components are also estimated by this program and method agreement was calculated according to the principles of Streiner & Norman[38]. Decision analysis used in Generalizability Theory resulted in the estimated method agreement with the categorized GS of a randomly chosen therapist (out of 10 per group). The agreement among therapist and the mutual agreement between disciplines was also calculated. This method agreement can be interpreted as a multivariate Intraclass Correlation Coefficient (ICC) as well as a multivariate Kappa[39,40]. Results can be between '0' i.e.: no method agreement at all and '1' i.e.: perfect method agreement. A method agreement of 0.80 is defined as acceptable[40]. Throughout the text, a p-value of less than 0.05 is considered statistically significant.

## Results

**Figure 2:**
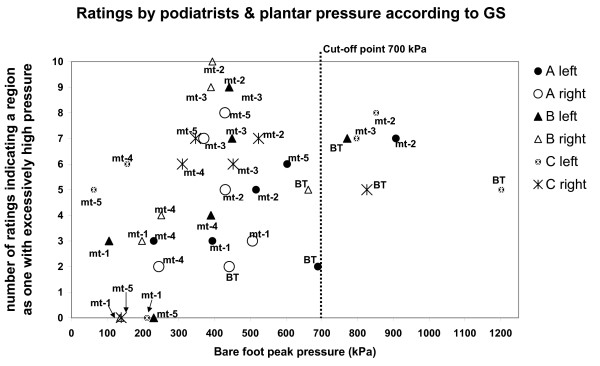
**Ratings by podiatrists**. The distribution of ratings of podiatrists who indicated a region as one with excessively high pressure, against the plantar pressure measured with the pressure platform (Gold Standard).

**Figure 3:**
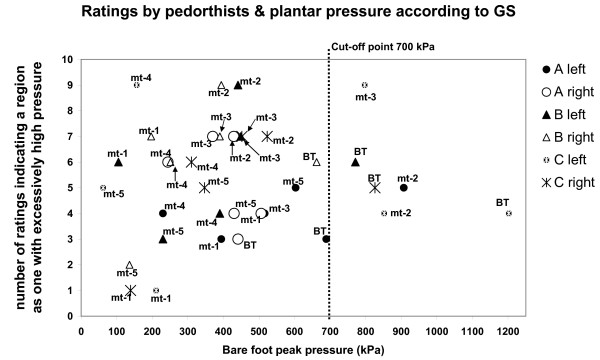
**Ratings by pedorthists**. The distribution of ratings of pedorthists who indicated a region as one with excessively high pressure, against the plantar pressure measured with the pressure platform (Gold Standard).

**Figure 4:**
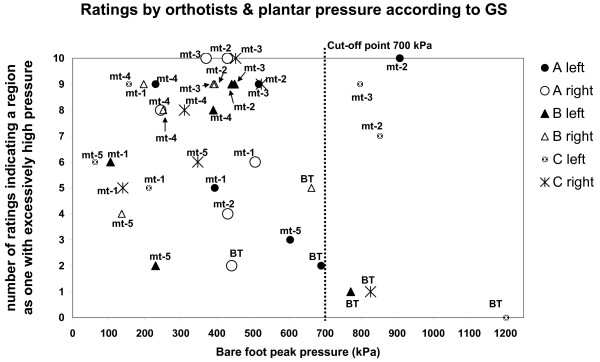
**Ratings by orthotists**. The distribution of ratings of orthotists who indicated a region as one with excessively high pressure, against the plantar pressure measured with the pressure platform (Gold Standard).

The frequency distribution of regions with excessively high pressure, as rated by the therapists, against the plantar pressure measured with the pressure platform (GS) are presented per discipline in figures 2 to 4. Table 1 presents the background characteristics of the patients included in the study. Table 2 provides the pressure platform (GS) dynamic bare foot peak pressure measurements (in kPa) for each of the six forefoot regions and for both feet in each patient. High plantar pressure measurements (>700 kPa) have been marked with boxes.

The observed proportions of elevated plantar pressure according to the GS and to the ratings per discipline, averaged over three patients (both feet and all six regions) are presented in Table 3. The differences in proportions of elevated plantar pressures between the GS and the ratings per discipline show significant discrepancies (all p-values < .05). On the average, one can see an underestimation in the big toe region and an overestimation in the other regions when compared to the GS. The standard deviations indicate large variations around the point estimate. For example, the proportion of elevated plantar pressure per location according to the GS is determined by the number of regions with observed elevated pressure to all regions for that location: for BT, three of the six regions (three patients both feet) had elevated pressure i.e. 3:6 = 0.50. The proportion for BT based on ratings by the three disciplines was 0.36, indicating an underestimation. Regions with elevated plantar pressure were frequently underrated: 35% of the podiatrists, 45% of the pedorthists and 53% orthotists, did not indicate the pertaining regions as having high pressure, i.e. they scored falsely negative. Ratings of orthotists for high pressure under the BT were illustrative: right side of figure 4.

Table 4 presents the estimated within-groups method agreement ICCs between GS and ratings. This ICC is calculated for each of the three disciplines and for each of the six forefoot regions, i.e. taking into account variance between therapists and variance between ratings and GS, see appendix formula A1. The mechanism of this comprehensive measure of agreement can be clarified by table 5, where the components of variance used for the BT region per discipline are presented. Part A of table 5 is the numerator of formula A1, whereas part B contains the relevant variance components of the denominator. The ICC is calculated through A_total_/(A_total _+B_total_). For the BT region, one can see that orthotists disagree more with the GS than podiatrists and pedorthists (see the overall prevalence proportions in table 3). However, the ICC for the orthotist method agreement is somewhat higher (0.86) than the ones for podiatrists (0.77) and pedorthists (0.77).

The **P_atient _× M_ethod _component **which accounts for the variance between clinical judgment and GS, is for orthotists relatively large (34%) compared to the ones for podiatrists (15%) and pedorthists (20%), see also table 3. The within group variance, **P_atient _× T_herapist _component**, for orthotists is relatively small (3%) showing good inter-observer agreement compared to the within group variance of podiatrists (14%) and pedorthists (9%), table 5. This explains why the orthotist ICC is large despite the GS differences on overall prevalence proportion. However, when the inter-observer agreement is calculated over all regions and the **P_atient _× M_ethod _component **is excluded (formula A5), then the ICCs result in: 0.18 for orthotists, 0.13 for podiatrists and 0.04 for pedorthists.

The estimated method agreement (ICC) per discipline, is 0.67, 0.65 and 0.76 for podiatrists, pedorthists and orthotists respectively (table 4). These ICCs are averaged over both feet and over all regions: formula A3. The overall method agreement, calculated through formula A4, turns out to be 0.70. All these results are below the critical level for sufficient agreement (0.80) and it appears that there is no discipline superior to another. Regions in which an acceptable level of estimated method agreement with GS is reached are BT and mt-2, respectively 0.80 and 0.82: formula A2.

Table 6 shows the estimated mutual method agreement (ICC) between the three disciplines. The ICC in this table can be interpreted as a generalized multivariate kappa between ratings of two professional groups. Most of the estimated mutual agreement ICCs for the separate regions are below 0.50 (formula B1), which indicates that there was a low mutual agreement among therapists from different disciplines. Whereas, the mutual method agreement averaged over regions, calculated through formula B2, showed virtually no agreement.

## Discussion

We examined three professional groups of foot care specialist regarding their ability to identify locations with elevated plantar pressures. The proportions based on clinical ratings of the therapists, show rather worrisome dissimilarities with the criterion values obtained trough quantitative plantar pressure measurement (GS). On the average, plantar pressures in the big toe region were underestimated and those in the metatarsal regions were overestimated. The estimated method agreement of clinical judgement of plantar pressures with the GS was below a predominated acceptable level. This generalized measure of agreement takes into various sources of variation. If one solely look to the agreement among therapists, then the results are fairly poor. The estimated mutual agreements showed that there was a low mutual agreement between the professional groups studied, indicating a large variability between disciplines. The problem of professional variability for other aspects of foot care was shown in a previous report [15].

There is some discussion about the level of agreement for medical diagnosis by professionals in day-by-day care. We considered that identification of locations with elevated pressures is an important aspect in foot care decision-making. For example, in the diabetic foot accurate identification is essential to define the treatment for prevention of (re)ulceration. Over- and underestimating plantar pressure, i.e. false positive and false negative ratings respectively, may lead to neglect of harmful elevated pressures. False negative means that a region was indicated as one without excessively high pressure, while the actual peak pressure was above the criterion level i.e.: 700 kPa and vice versa for false positive. Off-loading strategies in e.g. foot orthoses therapy, is achieved by shifting the excessive load to adjacent areas[41]. As a consequence, the actual 'risk region' could be exposed to a higher load and this could be disastrous for patients with insensate feet.

For identification of plantar locations with excessive pressure, most therapists used a footprint device, complementary to their physical examination. These ink print devices are based on 'the Harris and Beath foot printing technique'[42] and consist of a rubber mat with crosswise ridges. It is claimed that from the shade in the ink patterns, a semi-quantitative judgement about plantar pressures can be made[8,42]. Silvino demonstrated that ink print shades only could be distinguished below a pressure of 260 kPa, after which full ink blotting occurred and no further differentiation in plantar pressure could be made. Most normal, but especially harmful bare foot plantar pressures in e.g. diabetes and rheumatoid arthritis are much higher than 260 kPa. Therefore these ink print devices are no alternative for clinical quantitative plantar pressure measurement. Nonetheless, there was a large variety of methods used by the therapists in taking the footprints e.g. static versus dynamic measurements and semi – versus full weight bearing. These differences have a major influence on pressure patterns[43,44] and this could explain the misidentification of the elevated pressure under the big toe. For example, Cavanagh found that the toes were minimally involved in the static load bearing of the foot: less than 4%[27]. In contrast, the highest dynamic pressures were found under the metatarsal heads and the big toe[24,26,28,45]. Further, it was found that dynamic plantar pressure measurements show high-risk areas in diabetic patients more effectively than static pressure measurements[46].

We have used a cut-off point of 700 kPa for categorising elevated and non-elevated pressures. This arbitrary value is frequently cited as a critical threshold in foot disorders. If we lower the cut-off point to 600 kPa, then the GS proportion of elevated pressure in the big toe region would increase. This is illustrated by a shift of the cut-off point from 700 kPa to 600 kPa in figures 2 to 4. As a result now five BT regions are categorized as elevated, instead of three regions. This leads to a larger underestimation or false-negative rate: 5:6 = 0.83 versus 0.36. Not until 500 kPa, the results will change in favour of the therapist's ratings for the mt-1 to mt-3 regions (table 2 and figures 2 to 4). Regarding the condition of the patients who participated in this study, we consider a cut-off point 500 kPa as inappropriate. It is still unclear which parameter is most important i.e. the peak pressure or the pressure-time integral, and what can be seen as abnormal values for a specific foot condition[17]. Although, it is widely accepted that 'relatively' elevated foot pressure is an important factor in development of foot complications [16-23]. In the present study three patients with metatarsalgia were evaluated. Studies in patients with other foot pathologies are warranted to put our findings in a wider perspective.

We understand that the method of analysis used in this paper is difficult to comprehend. However, the implication of the results are crystal clear. This study showed that the clinical process for identification of elevated plantar pressure performed by the therapists appears to be insufficient. Aspects of physical examination, clinical reasoning and techniques for elevated plantar pressure screening have to be re-evaluated to improve this clinical process. On the long road ahead, the first step should be an inventory of possible valid methods for plantar pressure screening. Second: a detailed study has to be performed to find a clinical method or a combination of methods and techniques, which can most accurately identify locations with elevated pressure. Third: after validation and standardisation, the best method should be trained and implemented as a standard guideline.

In absence of a useful clinical screening method, quantitative plantar pressure measurement is a valuable alternative for screening. Although the last years, the price of this equipment has decreased and easy-to-use soft- and hardware has become available, plantar pressure measurement is not a standard facility in foot care practices. The financial investment for purchase and exploit of this equipment seems relatively small compared to the health benefits.

## Conclusion

Exact identification of locations with elevated plantar pressure through clinical evaluation is difficult: pressures in the big toe region tend to be underestimated and are likely to be overestimated in the metatarsal regions. Apart from being neglected, inaccurate identification of elevated pressure could result in mistreatment. There was a poor agreement between the therapists studied. There appears to be no relevant difference in the proficiency to distinguish regions with elevated plantar pressure between podiatrists, pedorthists and orthotists. The process of clinical plantar pressure screening has to be re-evaluated. The results of this study point towards the merit of quantitative plantar pressure measurement for clinical practice.

## Competing interests

The author(s) declare that they have no competing interests.

## Authors' contributions

All authors designed the study. NG collected and prepared the data. NG, PL and FN analyzed the data. All authors interpreted and discussed the data. NG, PL, FN and GW drafted the manuscript. NA, FN and PL revised the manuscript. All authors read and approved the final manuscript.

## Appendix

**formula's **where: σ^2 ^= estimated variance component, P = # patient, M = method, G = group, T = # therapist, R = region, S = side, and e designates the ultimate 'error' σ^2 ^component in the model specified.

Table 4

**Group- and Region specific formula for Method Agreement – upper left part of **Table 4.

**Model: **per Region and Group: Therapist × Method × (Side within Patient).

A1=σP2+σPM2+σPT2+σS:P2+σMS:P2+σTS:P2same as numerator+σPMT2+σMTS:P2,e
 MathType@MTEF@5@5@+=feaafiart1ev1aaatCvAUfKttLearuWrP9MDH5MBPbIqV92AaeXatLxBI9gBaebbnrfifHhDYfgasaacH8akY=wiFfYdH8Gipec8Eeeu0xXdbba9frFj0=OqFfea0dXdd9vqai=hGuQ8kuc9pgc9s8qqaq=dirpe0xb9q8qiLsFr0=vr0=vr0dc8meaabaqaciaacaGaaeqabaqabeGadaaakeaacqqGbbqqcqaIXaqmcqGH9aqpdaWcaaqaaiabeo8aZnaaDaaaleaacqqGqbauaeaacqaIYaGmaaGccqGHRaWkcqaHdpWCdaqhaaWcbaGaeeiuaaLaeeyta0eabaGaeGOmaidaaOGaey4kaSIaeq4Wdm3aa0baaSqaaiabbcfaqjabbsfaubqaaiabikdaYaaakiabgUcaRiabeo8aZnaaDaaaleaacqqGtbWucqqG6aGocqqGqbauaeaacqaIYaGmaaGccqGHRaWkcqaHdpWCdaqhaaWcbaGaeeyta0Kaee4uamLaeeOoaOJaeeiuaafabaGaeGOmaidaaOGaey4kaSIaeq4Wdm3aa0baaSqaaiabbsfaujabbofatjabbQda6iabbcfaqbqaaiabikdaYaaaaOqaaiabbohaZjabbggaHjabb2gaTjabbwgaLjabbccaGiabbggaHjabbohaZjabbccaGiabb6gaUjabbwha1jabb2gaTjabbwgaLjabbkhaYjabbggaHjabbsha0jabb+gaVjabbkhaYjabgUcaRiabeo8aZnaaDaaaleaacqqGqbaucqqGnbqtcqqGubavaeaacqaIYaGmaaGccqGHRaWkcqaHdpWCdaqhaaWcbaGaeeyta0KaeeivaqLaee4uamLaeeOoaOJaeeiuaafabaGaeGOmaidaaOGaeiilaWIaeeyzaugaaaaa@80B7@

**Region-specific formula for Method Agreement – right totals column in **Table 4.

**Model **per Region: Method × (Therapist within Group) × (Side within Patient).

A2=σP2+σPM2+σPG2+σPGM2+σGS:P2+σPT:G2+σTS:PG2+σSM:P2+σGSM:P2same as numerator+σPMT:G2+σTSM:PG2,e
 MathType@MTEF@5@5@+=feaafiart1ev1aaatCvAUfKttLearuWrP9MDH5MBPbIqV92AaeXatLxBI9gBaebbnrfifHhDYfgasaacH8akY=wiFfYdH8Gipec8Eeeu0xXdbba9frFj0=OqFfea0dXdd9vqai=hGuQ8kuc9pgc9s8qqaq=dirpe0xb9q8qiLsFr0=vr0=vr0dc8meaabaqaciaacaGaaeqabaqabeGadaaakeaacqqGbbqqcqaIYaGmcqGH9aqpdaWcaaqaaiabeo8aZnaaDaaaleaacqqGqbauaeaacqaIYaGmaaGccqGHRaWkcqaHdpWCdaqhaaWcbaGaeeiuaaLaeeyta0eabaGaeGOmaidaaOGaey4kaSIaeq4Wdm3aa0baaSqaaiabbcfaqjabbEeahbqaaiabikdaYaaakiabgUcaRiabeo8aZnaaDaaaleaacqqGqbaucqqGhbWrcqqGnbqtaeaacqaIYaGmaaGccqGHRaWkcqaHdpWCdaqhaaWcbaGaee4raCKaee4uamLaeeOoaOJaeeiuaafabaGaeGOmaidaaOGaey4kaSIaeq4Wdm3aa0baaSqaaiabbcfaqjabbsfaujabbQda6iabbEeahbqaaiabikdaYaaakiabgUcaRiabeo8aZnaaDaaaleaacqqGubavcqqGtbWucqqG6aGocqqGqbaucqqGhbWraeaacqaIYaGmaaGccqGHRaWkcqaHdpWCdaqhaaWcbaGaee4uamLaeeyta0KaeeOoaOJaeeiuaafabaGaeGOmaidaaOGaey4kaSIaeq4Wdm3aa0baaSqaaiabbEeahjabbofatjabb2eanjabbQda6iabbcfaqbqaaiabikdaYaaaaOqaaiabbohaZjabbggaHjabb2gaTjabbwgaLjabbccaGiabbggaHjabbohaZjabbccaGiabb6gaUjabbwha1jabb2gaTjabbwgaLjabbkhaYjabbggaHjabbsha0jabb+gaVjabbkhaYjabgUcaRiabeo8aZnaaDaaaleaacqqGqbaucqqGnbqtcqqGubavcqqG6aGocqqGhbWraeaacqaIYaGmaaGccqGHRaWkcqaHdpWCdaqhaaWcbaGaeeivaqLaee4uamLaeeyta0KaeeOoaOJaeeiuaaLaee4raCeabaGaeGOmaidaaOGaeiilaWIaeeyzaugaaaaa@9EA0@

**Group-specific formula for Method Agreement-bottom totals row in **Table 4.

**Model **per Group: Therapist × Method × [(Region × Side) within Patient]

A3=σP2+σPM2+σPT2+σR:P2+σS:P2+σTR:P2+σTS:P2+σRS:P2+σRM:P2+σSM:P2+σSM:P2+σTRS:P2+σRSM:P2same as numerator+σPMT2+σTRM:P2+σTSM:P2+σTRSM:P2,e
 MathType@MTEF@5@5@+=feaafiart1ev1aaatCvAUfKttLearuWrP9MDH5MBPbIqV92AaeXatLxBI9gBaebbnrfifHhDYfgasaacH8akY=wiFfYdH8Gipec8Eeeu0xXdbba9frFj0=OqFfea0dXdd9vqai=hGuQ8kuc9pgc9s8qqaq=dirpe0xb9q8qiLsFr0=vr0=vr0dc8meaabaqaciaacaGaaeqabaqabeGadaaakeaacqqGbbqqcqaIZaWmcqGH9aqpdaWcaaqaaiabeo8aZnaaDaaaleaacqqGqbauaeaacqaIYaGmaaGccqGHRaWkcqaHdpWCdaqhaaWcbaGaeeiuaaLaeeyta0eabaGaeGOmaidaaOGaey4kaSIaeq4Wdm3aa0baaSqaaiabbcfaqjabbsfaubqaaiabikdaYaaakiabgUcaRiabeo8aZnaaDaaaleaacqqGsbGucqqG6aGocqqGqbauaeaacqaIYaGmaaGccqGHRaWkcqaHdpWCdaqhaaWcbaGaee4uamLaeeOoaOJaeeiuaafabaGaeGOmaidaaOGaey4kaSIaeq4Wdm3aa0baaSqaaiabbsfaujabbkfasjabbQda6iabbcfaqbqaaiabikdaYaaakiabgUcaRiabeo8aZnaaDaaaleaacqqGubavcqqGtbWucqqG6aGocqqGqbauaeaacqaIYaGmaaGccqGHRaWkcqaHdpWCdaqhaaWcbaGaeeOuaiLaee4uamLaeeOoaOJaeeiuaafabaGaeGOmaidaaOGaey4kaSIaeq4Wdm3aa0baaSqaaiabbkfasjabb2eanjabbQda6iabbcfaqbqaaiabikdaYaaakiabgUcaRiabeo8aZnaaDaaaleaacqqGtbWucqqGnbqtcqqG6aGocqqGqbauaeaacqaIYaGmaaGccqGHRaWkcqaHdpWCdaqhaaWcbaGaee4uamLaeeyta0KaeeOoaOJaeeiuaafabaGaeGOmaidaaOGaey4kaSIaeq4Wdm3aa0baaSqaaiabbsfaujabbkfasjabbofatjabbQda6iabbcfaqbqaaiabikdaYaaakiabgUcaRiabeo8aZnaaDaaaleaacqqGsbGucqqGtbWucqqGnbqtcqqG6aGocqqGqbauaeaacqaIYaGmaaaakeaacqqGZbWCcqqGHbqycqqGTbqBcqqGLbqzcqqGGaaicqqGHbqycqqGZbWCcqqGGaaicqqGUbGBcqqG1bqDcqqGTbqBcqqGLbqzcqqGYbGCcqqGHbqycqqG0baDcqqGVbWBcqqGYbGCcqGHRaWkcqaHdpWCdaqhaaWcbaGaeeiuaaLaeeyta0KaeeivaqfabaGaeGOmaidaaOGaey4kaSIaeq4Wdm3aa0baaSqaaiabbsfaujabbkfasjabb2eanjabbQda6iabbcfaqbqaaiabikdaYaaakiabgUcaRiabeo8aZnaaDaaaleaacqqGubavcqqGtbWucqqGnbqtcqqG6aGocqqGqbauaeaacqaIYaGmaaGccqGHRaWkcqaHdpWCdaqhaaWcbaGaeeivaqLaeeOuaiLaee4uamLaeeyta0KaeeOoaOJaeeiuaafabaGaeGOmaidaaOGaeiilaWIaeeyzaugaaaaa@CFC5@

**Overall formula for Method Agreement – bottom right, general total in **Table 4.

**Model **Method × (Therapist within Group) × [(Region × Side) within Patient]

A4=σP2+σPM2+σPG2+σS:P2+σR:P2+σPMG2+σMS:P2+σMR:P2+σGS:P2+σGR:P2+σSR:P2+σMGS:P2+σMGR:P2+σMSR:P2+σGSR:P2+σMGSR:P2+σPT:G2+σST:PG2+σRT:PG2+σRST:PG2same as numerator+σPMT:G2+σMTS:PG2+σMTR:PG2+σMTSR:PG2,e
 MathType@MTEF@5@5@+=feaafiart1ev1aaatCvAUfKttLearuWrP9MDH5MBPbIqV92AaeXatLxBI9gBaebbnrfifHhDYfgasaacH8akY=wiFfYdH8Gipec8Eeeu0xXdbba9frFj0=OqFfea0dXdd9vqai=hGuQ8kuc9pgc9s8qqaq=dirpe0xb9q8qiLsFr0=vr0=vr0dc8meaabaqaciaacaGaaeqabaqabeGadaaakeaacqqGbbqqcqaI0aancqGH9aqpdaWcaaqaauaabeqaceaaaeaacqaHdpWCdaqhaaWcbaGaeeiuaafabaGaeGOmaidaaOGaey4kaSIaeq4Wdm3aa0baaSqaaiabbcfaqjabb2eanbqaaiabikdaYaaakiabgUcaRiabeo8aZnaaDaaaleaacqqGqbaucqqGhbWraeaacqaIYaGmaaGccqGHRaWkcqaHdpWCdaqhaaWcbaGaee4uamLaeeOoaOJaeeiuaafabaGaeGOmaidaaOGaey4kaSIaeq4Wdm3aa0baaSqaaiabbkfasjabbQda6iabbcfaqbqaaiabikdaYaaakiabgUcaRiabeo8aZnaaDaaaleaacqqGqbaucqqGnbqtcqqGhbWraeaacqaIYaGmaaGccqGHRaWkcqaHdpWCdaqhaaWcbaGaeeyta0Kaee4uamLaeeOoaOJaeeiuaafabaGaeGOmaidaaOGaey4kaSIaeq4Wdm3aa0baaSqaaiabb2eanjabbkfasjabbQda6iabbcfaqbqaaiabikdaYaaakiabgUcaRiabeo8aZnaaDaaaleaacqqGhbWrcqqGtbWucqqG6aGocqqGqbauaeaacqaIYaGmaaGccqGHRaWkcqaHdpWCdaqhaaWcbaGaee4raCKaeeOuaiLaeeOoaOJaeeiuaafabaGaeGOmaidaaOGaey4kaScabaGaeq4Wdm3aa0baaSqaaiabbofatjabbkfasjabbQda6iabbcfaqbqaaiabikdaYaaakiabgUcaRiabeo8aZnaaDaaaleaacqqGnbqtcqqGhbWrcqqGtbWucqqG6aGocqqGqbauaeaacqaIYaGmaaGccqGHRaWkcqaHdpWCdaqhaaWcbaGaeeyta0Kaee4raCKaeeOuaiLaeeOoaOJaeeiuaafabaGaeGOmaidaaOGaey4kaSIaeq4Wdm3aa0baaSqaaiabb2eanjabbofatjabbkfasjabbQda6iabbcfaqbqaaiabikdaYaaakiabgUcaRiabeo8aZnaaDaaaleaacqqGhbWrcqqGtbWucqqGsbGucqqG6aGocqqGqbauaeaacqaIYaGmaaGccqGHRaWkcqaHdpWCdaqhaaWcbaGaeeyta0Kaee4raCKaee4uamLaeeOuaiLaeeOoaOJaeeiuaafabaGaeGOmaidaaOGaey4kaSIaeq4Wdm3aa0baaSqaaiabbcfaqjabbsfaujabbQda6iabbEeahbqaaiabikdaYaaakiabgUcaRiabeo8aZnaaDaaaleaacqqGtbWucqqGubavcqqG6aGocqqGqbaucqqGhbWraeaacqaIYaGmaaGccqGHRaWkcqaHdpWCdaqhaaWcbaGaeeOuaiLaeeivaqLaeeOoaOJaeeiuaaLaee4raCeabaGaeGOmaidaaOGaey4kaSIaeq4Wdm3aa0baaSqaaiabbkfasjabbofatjabbsfaujabbQda6iabbcfaqjabbEeahbqaaiabikdaYaaaaaaakeaacqqGZbWCcqqGHbqycqqGTbqBcqqGLbqzcqqGGaaicqqGHbqycqqGZbWCcqqGGaaicqqGUbGBcqqG1bqDcqqGTbqBcqqGLbqzcqqGYbGCcqqGHbqycqqG0baDcqqGVbWBcqqGYbGCcqGHRaWkcqaHdpWCdaqhaaWcbaGaeeiuaaLaeeyta0KaeeivaqLaeeOoaOJaee4raCeabaGaeGOmaidaaOGaey4kaSIaeq4Wdm3aa0baaSqaaiabb2eanjabbsfaujabbofatjabbQda6iabbcfaqjabbEeahbqaaiabikdaYaaakiabgUcaRiabeo8aZnaaDaaaleaacqqGnbqtcqqGubavcqqGsbGucqqG6aGocqqGqbaucqqGhbWraeaacqaIYaGmaaGccqGHRaWkcqaHdpWCdaqhaaWcbaGaeeyta0KaeeivaqLaee4uamLaeeOuaiLaeeOoaOJaeeiuaaLaee4raCeabaGaeGOmaidaaOGaeiilaWIaeeyzaugaaaaa@164A@

Formula for agreement among therapists

**Model **Therapist × (Side within Patient)

A5=σP2+σS:P2σP2+σS:P2+σPT2+σTS:P2,e
 MathType@MTEF@5@5@+=feaafiart1ev1aaatCvAUfKttLearuWrP9MDH5MBPbIqV92AaeXatLxBI9gBaebbnrfifHhDYfgasaacH8akY=wiFfYdH8Gipec8Eeeu0xXdbba9frFj0=OqFfea0dXdd9vqai=hGuQ8kuc9pgc9s8qqaq=dirpe0xb9q8qiLsFr0=vr0=vr0dc8meaabaqaciaacaGaaeqabaqabeGadaaakeaacqqGbbqqcqaI1aqncqGH9aqpdaWcaaqaaiabeo8aZnaaDaaaleaacqqGqbauaeaacqaIYaGmaaGccqGHRaWkcqaHdpWCdaqhaaWcbaGaee4uamLaeeOoaOJaeeiuaafabaGaeGOmaidaaaGcbaGaeq4Wdm3aa0baaSqaaiabbcfaqbqaaiabikdaYaaakiabgUcaRiabeo8aZnaaDaaaleaacqqGtbWucqqG6aGocqqGqbauaeaacqaIYaGmaaGccqGHRaWkcqaHdpWCdaqhaaWcbaGaeeiuaaLaeeivaqfabaGaeGOmaidaaOGaey4kaSIaeq4Wdm3aa0baaSqaaiabbsfaujabbofatjabbQda6iabbcfaqbqaaiabikdaYaaakiabcYcaSiabbwgaLbaaaaa@56C4@

Table 6

**Two Groups- and Region specific formula for mutual Agreement: upper part of **Table 6.

**Model **per Region and for two Groups: (Therapist within Group) × (Side within Patient)

B1=σP2+σPG2+σS:P2+σGS:P2same as numerator+σPT:G2+σTS:PG2,e
 MathType@MTEF@5@5@+=feaafiart1ev1aaatCvAUfKttLearuWrP9MDH5MBPbIqV92AaeXatLxBI9gBaebbnrfifHhDYfgasaacH8akY=wiFfYdH8Gipec8Eeeu0xXdbba9frFj0=OqFfea0dXdd9vqai=hGuQ8kuc9pgc9s8qqaq=dirpe0xb9q8qiLsFr0=vr0=vr0dc8meaabaqaciaacaGaaeqabaqabeGadaaakeaacqqGcbGqcqaIXaqmcqGH9aqpdaWcaaqaaiabeo8aZnaaDaaaleaacqqGqbauaeaacqaIYaGmaaGccqGHRaWkcqaHdpWCdaqhaaWcbaGaeeiuaaLaee4raCeabaGaeGOmaidaaOGaey4kaSIaeq4Wdm3aa0baaSqaaiabbofatjabbQda6iabbcfaqbqaaiabikdaYaaakiabgUcaRiabeo8aZnaaDaaaleaacqqGhbWrcqqGtbWucqqG6aGocqqGqbauaeaacqaIYaGmaaaakeaacqqGZbWCcqqGHbqycqqGTbqBcqqGLbqzcqqGGaaicqqGHbqycqqGZbWCcqqGGaaicqqGUbGBcqqG1bqDcqqGTbqBcqqGLbqzcqqGYbGCcqqGHbqycqqG0baDcqqGVbWBcqqGYbGCcqGHRaWkcqaHdpWCdaqhaaWcbaGaeeiuaaLaeeivaqLaeeOoaOJaee4raCeabaGaeGOmaidaaOGaey4kaSIaeq4Wdm3aa0baaSqaaiabbsfaujabbofatjabbQda6iabbcfaqjabbEeahbqaaiabikdaYaaakiabcYcaSiabbwgaLbaaaaa@7314@

**Two Groups specific formula for mutual Agreement: bottom totals row in **Table 6.

**Model **for two Groups: (Therapist within Group) × [(Region × Side) within Patient]

B2=σP2+σPG2+σS:P2+σR:P2+σGS:P2+σGR:P2+σGSR:P2+σSR:P2same as numerator+σPT2+σST:PG2+σRT:PG2+σRST:PG2,e
 MathType@MTEF@5@5@+=feaafiart1ev1aaatCvAUfKttLearuWrP9MDH5MBPbIqV92AaeXatLxBI9gBaebbnrfifHhDYfgasaacH8akY=wiFfYdH8Gipec8Eeeu0xXdbba9frFj0=OqFfea0dXdd9vqai=hGuQ8kuc9pgc9s8qqaq=dirpe0xb9q8qiLsFr0=vr0=vr0dc8meaabaqaciaacaGaaeqabaqabeGadaaakeaacqqGcbGqcqaIYaGmcqGH9aqpdaWcaaqaaiabeo8aZnaaDaaaleaacqqGqbauaeaacqaIYaGmaaGccqGHRaWkcqaHdpWCdaqhaaWcbaGaeeiuaaLaee4raCeabaGaeGOmaidaaOGaey4kaSIaeq4Wdm3aa0baaSqaaiabbofatjabbQda6iabbcfaqbqaaiabikdaYaaakiabgUcaRiabeo8aZnaaDaaaleaacqqGsbGucqqG6aGocqqGqbauaeaacqaIYaGmaaGccqGHRaWkcqaHdpWCdaqhaaWcbaGaee4raCKaee4uamLaeeOoaOJaeeiuaafabaGaeGOmaidaaOGaey4kaSIaeq4Wdm3aa0baaSqaaiabbEeahjabbkfasjabbQda6iabbcfaqbqaaiabikdaYaaakiabgUcaRiabeo8aZnaaDaaaleaacqqGhbWrcqqGtbWucqqGsbGucqqG6aGocqqGqbauaeaacqaIYaGmaaGccqGHRaWkcqaHdpWCdaqhaaWcbaGaee4uamLaeeOuaiLaeeOoaOJaeeiuaafabaGaeGOmaidaaaGcbaGaee4CamNaeeyyaeMaeeyBa0MaeeyzauMaeeiiaaIaeeyyaeMaee4CamNaeeiiaaIaeeOBa4MaeeyDauNaeeyBa0MaeeyzauMaeeOCaiNaeeyyaeMaeeiDaqNaee4Ba8MaeeOCaiNaey4kaSIaeq4Wdm3aa0baaSqaaiabbcfaqjabbsfaubqaaiabikdaYaaakiabgUcaRiabeo8aZnaaDaaaleaacqqGtbWucqqGubavcqqG6aGocqqGqbaucqqGhbWraeaacqaIYaGmaaGccqGHRaWkcqaHdpWCdaqhaaWcbaGaeeOuaiLaeeivaqLaeeOoaOJaeeiuaaLaee4raCeabaGaeGOmaidaaOGaey4kaSIaeq4Wdm3aa0baaSqaaiabbkfasjabbofatjabbsfaujabbQda6iabbcfaqjabbEeahbqaaiabikdaYaaakiabcYcaSiabbwgaLbaaaaa@A5E1@

## Pre-publication history

The pre-publication history for this paper can be accessed here:



## Figures and Tables

**Table 1:** Patient characteristics

Patient	**A**		**B**		**C**	
Gender	female		Female		male	

Age (yr)	60		61		37	
Weight (kg)	105		73		82	
Body length (cm)	178		154		181	
Systemic diseases					Arthritis psoriatica	

	left	right	left	right	left	right

**Structural classification**						
pes plano valgus	**x**	**x**				
calcaneus valgus					**x**	**x**
hallux valgus			**x**	**x**		
bunion			**x**	**x**		
claw toes					**x**	**x**
**Specific diagnosis**						
Metatarsalgia	**x**	**x**	**x**	**x**	**x**	**x**
Plantar fasciitis		**x**				
MTP-1 joint Extension	50°	50°	50°	35°	55°	60°
MTP-1 joint Flexion	45°	40°	30°	35°	40°	45°

Yr = years, kg = kilogram, cm = centimetre, MTP = metatarsophalangeal

**Table 2:** 'Gold standard': dynamic bare foot peak pressures for patient A, B and C (kPa)

	**A left**	**A right**	**B left**	**B right**	**C left**	**C right**
**BT**	689	441	**771**	662	**1202**	**826**
**mt1**	394	506	105	197	211	139
**mt2**	**907**	429	441	394	**852**	523
**mt3**	516	370	449	390	**797**	452
**mt4**	230	244	390	251	156	310
**mt5**	603	430	230	136	62	347

Peak pressures greater than 700 kPa are outlined and bold printed. BT = big toe, mt = metatarsal

**Table 3:** Observed proportions of elevated plantar pressure according to the GS and to the ratings per discipline

**Region**	**GS**	**Podiatrist**	**Pedorthist**	**Orthotist**	**Over all disciplines**
**BT**	0.50	0.43 ± 0.50	0.45 ± 0.50	0.18 ± 0.39	0.36* ± 0.48
**mt-1**	0.00	0.20 ± 0.40	0.37 ± 0.48	0.60 ± 0.49	0.39** ± 0.49
**mt-2**	0.33	0.82 ± 0.39	0.68 ± 0.47	0.90 ± 0.30	0.80** ± 0.40
**mt-3**	0.17	0.68 ± 0.47	0.68 ± 0.47	0.93 ± 0.25	0.77** ± 0.44
**mt-4**	0.00	0.42 ± 0.49	0.58 ± 0.49	0.83 ± 0.37	0.61** ± 0.49
**mt-5**	0.00	0.38 ± 0.49	0.40 ± 0.49	0.42 ± 0.49	0.40** ± 0.49

**Total**	**0.17**	**0.49 **± 0.50	0.52* ± 0.50	0.64** ± 0.48	0.55** ± 0.50

The proportion of elevated plantar pressure per region according to the GS is determined by the number of regions with observed elevated pressure to all regions for that location: for example, three of the six BT regions (three patients both feet) had elevated pressure i.e. 3:6 = 0.50. For each region, the ratings per discipline were averaged over both feet of the patients. BT = big toe, mt = metatarsal. ± standard deviation. * = p value < .05, ** = p value < .005

**Table 4:** Estimated within group Method Agreement (ICC)

**Region**	**Podiatrist**	**Pedorthist**	**Orthotist**	**Averaged total**
**BT**	0.77	0.77	0.86	**0.80**
**mt-1**	0.38	0.68	0.36	**0.42**
**mt-2**	0.82	0.78	0.87	**0.82**
**mt-3**	0.64	0.62	0.83	**0.71**
**mt-4**	0.36	0.39	0.33	**0.36**
**mt-5**	0.56	0.33	0.39	**0.48**

**Averaged total**	**0.67**	**0.65**	**0.76**	**0.70**

Estimated Method Agreement (ICC) between so-called Gold Standard and subjective professional judgment of elevated plantar pressure in regions of both feet, specified for type of therapist and/or foot region. mt = metatarsal, BT = big toe

**Table 5:** Components of Variance used for calculation of the BT region Method Agreement ICCs in table 4

**Part A**									
	**Podiatrist**			**Pedorthist**			**Orthotist**		
**Source**	**σ^2^**	**%**	**SE**	**σ^2^**	**%**	**SE**	**σ^2^**	**%**	**SE**
**P**	0.1065	25	0.078	0.0831	18	0.061	0.0458	9	0.034
**P × M**	0.0630	15	0.051	0.0912	20	0.070	0.1667	34	0.121
**P × T**	0.0593	14	0.014	0.0407	9	0.012	0.0139	3	0.006
**S within P**	0.0435	10	0.038	0.0377	8	0.027	0.0188	4	0.010
**M × S within P**	0.0519	12	0.034	0.0815	18	0.053	0.1611	33	0.104
**T × S within P**	0.0074	2	0.002	0.0134	3	0.004	0.0153	3	0.004
**Total**	**0.3315**			**0.3477**			**0.4215**		

**Part B**									
	**Podiatrist**			**Pedorthist**			**Orthotist**		
	**σ^2^**	**%**	**SE**	**σ^2^**	**%**	**SE**	**σ^2^**	**%**	**SE**

**P × M × T**	0.0870	20	0.028	0.0755	17	0.024	0.0375	8	0.012
**M × T × S within Pe**	0.0148	3	0.004	0.0269	6	0.007	0.0306	6	0.008
**Total**	**0.1019**			**0.1023**			**0.0681**		

Total percentage is the summation of part A and B. **σ^2 ^**= estimated variance component, **P **= patient, **M **= method, **G **= group, **T **= therapist, **S **= side, and **e **designates the ultimate 'error' σ^2 ^component in the model specified. SE = standard error, BT = big toe

**Table 6:** Estimated mutual Agreement (ICC)

**Region**	**Podiatrist-Pedorthist**	**Podiatrist-Orthotist**	**Pedorthist-Orthotist**
**BT**	0.53	0.59	0.32
**mt-1**	0.70	0.37	0.64
**mt-2**	0.44	0.17	0.48
**mt-3**	0.06	0.00	0.00
**mt-4**	0.36	0.01	0.01
**mt-5**	0.66	0.78	0.30

**Total**	**0.01**	**0.000**	**0.000**

Estimated mutual Agreement (ICC) of plantar peak pressure of one therapist out of 10 between two out of three disciplines (podiatrists, pedorthists and orthotists) and regions of the forefoot averaged over three patients (both feet). Results are between 0.000 (absolutely no mutual agreement) and 1.000 perfect mutual agreement).
